# Nature-Based Interventions in the UK: A Mixed Methods Study Exploring Green Prescribing for Promoting the Mental Wellbeing of Young Pregnant Women

**DOI:** 10.3390/ijerph20206921

**Published:** 2023-10-13

**Authors:** Gina Sands, Holly Blake, Tim Carter, Helen Spiby

**Affiliations:** 1 School of Health Sciences, University of Nottingham, Nottingham NG7 2HA, UK; holly.blake@nottingham.ac.uk (H.B.); tim.carter@nottingham.ac.uk (T.C.); helen.spiby@nottingham.ac.uk (H.S.); 2 NIHR Nottingham Biomedical Research Centre, Nottingham NG7 2UH, UK

**Keywords:** green prescribing, nature-based interventions, pregnancy, young women, social prescribing, green space, mental health, wellbeing

## Abstract

Green prescribing is gaining in popularity internationally for the promotion of mental wellbeing. However, the evidence base is limited, particularly in young pregnant women, a population with known risk factors for anxiety and depression. The aim of this mixed-methods study was to provide insights into the availability, processes, and suitability of nature-based interventions for young pregnant women. First, an online mapping survey of nature-based activities in the East Midlands region of the United Kingdom (UK) was undertaken. Second, focus groups (*n* = 6) were conducted with nature activity providers and young mothers (*n* = 11). This study found there were many diverse nature-based activities available to promote mental wellbeing. The organisational challenges highlighted include a lack of sufficient funding for service provision and disappointing experiences with some green prescribing programmes. The young women felt that nature-based activities helped to promote their mental wellbeing, and also offered an opportunity for social support. The facilitators, such as having detailed information and being accompanied to initial sessions to ease anxieties, were found to maximise the women’s engagement with nature-based interventions. This study provides new perspectives on nature-based interventions from service providers and young women. Findings on the organisational barriers and facilitators to delivering interventions will inform the design of much needed future experimental research.

## 1. Introduction

There is increasing evidence that access and connection to nature is beneficial to mental health and general wellbeing [1,2,3,4,5] and mitigates socio-economic health inequalities [6]. There is a growing movement of social and green prescribing worldwide as healthcare commissioners and public health bodies acknowledge the associated health [7] and economic benefits [8]. Social prescribing is when health professionals refer patients to support in the community to improve their health and wellbeing. Green prescribing is the process of healthcare professionals referring a patient to perform nature-based activities (sometimes exercise) rather than prescribing medicine. Japan and New Zealand were among the first to offer green prescriptions as early as the 1980s. Japan has a long history of promoting public health through ‘shinrin yoku’ or ‘forest bathing’, but as with other types of nature-based interventions, the evidence base mostly consists of lower quality studies with a high-medium risk of bias [9]. A recent meta-analysis (2021) demonstrated a large and significant effect for nature-based interventions improving depressive mood and reducing anxiety, however, no studies focused on pregnant women [1].

More women in the United Kingdom (UK) are presenting with mental ill-health than ever before and rates continue to rise whilst rates in men have remained stable over the last 20 years [10]. Young women between the ages of 16–25 years are particularly vulnerable, with a quarter of young women now reporting symptoms of mental ill-health [10,11]. This risk increases further for pregnant and postnatal women, as perinatal and postnatal mental health disorders are some of the most common co-morbidities of pregnancy [12]. Mental ill-health during pregnancy has a devastating impact on women’s quality of life, relationships, and attachment with their child [13]. Maternal mental health problems also impact child health such as adverse birth outcomes [13,14], and emotional, behavioural, and developmental issues in childhood [13,15,16]. Recent reviews have reported the prevalence of antenatal and postnatal anxiety at 15–20% and 10%, respectively [17,18,19], with 17% of mothers experiencing postnatal depression [20]. Young pregnant women are particularly vulnerable to poor mental health and wellbeing [17,21]. Antenatal anxiety is significantly associated with young age [17], and adolescents have an increased risk of becoming depressed during [17,21] and after [21] pregnancy. Teenage pregnancy is also associated with a higher risk of depression and a lower Apgar score at birth [22]. There is substantial cost associated with this to the public sector, particularly to UK National Health Service (NHS) and social care. The combined costs of perinatal anxiety and depression are estimated to be £8500 per woman giving birth; 60% of these costs related to the adverse impact on children [15].

Observational studies suggest proximity to nature may have particular protective effects on mental health for females and lower income or lower education groups [23]. Pregnant women living in greener settings are 20% less likely to report symptoms of depression [24] and have a decreased risk for low birth weight after controlling for other factors [25]. Improving exposure to green space is suggested as a promising intervention for reducing risk of depression, particularly in disadvantaged groups [24]. However, connection with nature may be more important than exposure, and a framework to support this has been developed [26] with nature-connection interventions reporting significant improvements in mental health [27]. However, these encouraging findings do not focus on young pregnant women, therefore further research is required.

Lack of social support may increase perinatal anxiety and depression [17], but positive social support can improve symptoms and is important for wellbeing during maternity [17,21]. Indeed, increasing social support is well established to promote mental health and wellbeing [28,29,30]. In pregnancy, greater social support networks can protect against health outcomes, such as postnatal depression [31], and pregnancy complications in women with additional risk factors [30,32]. Additional social support in pregnancy has long term benefits on the psychosocial health of mothers and children’s health and development [33]. Group pregnancy care is also well received by young women and offers opportunities for learning and socialising amongst peers [34].

There is a critical need for new approaches to supporting the mental health of young women. This is particularly important during pregnancy where preventative mental health care is not currently offered, and treatment is often lacking or fragmented [35]. This study aimed to provide insights into the availability of nature-based group activities and whether these may be suitable for green prescribing interventions to promote mental health and wellbeing in young pregnant women.

## 2. Materials and Methods

A mixed methods approach was adopted consisting of (1) a mapping exercise of nature-based activity providers via an online survey, and (2) qualitative focus groups with women and nature-based activity providers. This study was designed to inform intervention design and further research in the East Midlands region of the UK; therefore, data collection was focused on this region.

### 2.1. Mapping Survey of Nature-Based Activities

A mapping exercise was completed to explore the service provision of nature-based activities available to pregnant young women across the East Midlands region of the UK. This followed the processes described in Price et al.’s ‘Seven steps to mapping health service provision’ [36] but was modified to focus on community rather than NHS services. The target service was defined as social, nature-based support and/or services to promote mental health and wellbeing which are available to (but not necessarily exclusively targeted at) young pregnant women aged 16–24 years. Inclusion criteria were as follows: social elements and based in nature (green or blue spaces); services which are inclusive to young pregnant women; services which promote mental health and wellbeing; and services located in the East Midlands of the UK (including local services offered by national organisations). The exclusion criteria were as follows: services targeting individuals with specific health issues, unless pregnancy related; services that aim to treat, rather than prevent, mental health issues; services which exclude young pregnant women; NHS services/support which are not based in the community or the target region.

Nature-based services and activities were identified through directories of voluntary services, social media, online search engines, and key informants such as green prescribing ‘test and learn sites’ operating in two areas. A snowballing technique was also employed in which identified organisations were asked if they had knowledge of other nature-based activities in the region. A short online survey of 27 items was designed by the research team to explore the nature-based activities on offer, organisational aspects (e.g., evaluation, impact, funding), and service user aspects (e.g., referrals, access). This was piloted for usability, relevance, and technical functionality with members of the Nottingham Maternity Research Network who had prior experience with community organisations. The survey (Supplementary File S1) was created using Microsoft Forms and the estimated completion time based on piloting was 10–15 min. There were a mix of mandatory questions and optional free text boxes for further comments. A convenience sampling approach was used by distributing the survey link via direct email to all nature-based activity services that had been identified (125 organisations), sharing via social media (Twitter and Facebook), and via local green prescribing networks. The survey was open for 11 weeks from April to July 2022. Organisations who did not respond to direct email were followed up by a second reminder email before the survey closed. Participants were advised how long the survey may take, that participation was voluntary, and that responses would remain anonymous unless they gave permission for details to be shared. No monetary incentives were offered. Categorical and free text data collected were combined to provide a descriptive summary of findings. The reporting of the survey is guided by the CHERRIES reporting checklist for online surveys [37].

### 2.2. Qualitative Focus Groups

#### 2.2.1. Young Women

Four focus groups were completed with women under 25 years old who had experience of pregnancy and birth at a younger age (total *n* = 11). These women were recruited through local services (e.g., children’s centre), social media, and word of mouth. Women were offered a £10 voucher to thank them for their participation. One of the focus groups was held in person, the others were held via MS Teams calls online. These focus groups were to explore interest in nature-based activities during pregnancy, particularly when pregnant at this relatively young age (16–25 years). Focus groups were facilitated by two researchers (GS, HS) over a one-hour period and followed a semi-structured topic guide. Written notes were taken during the focus groups, these were used to produce a thematic analysis.

#### 2.2.2. Nature Providers

Two focus groups with providers of nature-based activities in the East Midlands region of the UK (*n* = 6) were conducted in August 2022. The purpose of the focus groups was to explore the experiences of providing nature-based activities, engagement with green-prescribing, and suitability for young pregnant women. Participants were recruited from those who had completed the mapping survey and indicated that they would be happy to be contacted about further research. These were the founders of the organisation or those leading the nature-based activity programmes. Providers were offered a £10 voucher to thank them for their participation. Focus groups were facilitated by two researchers (GS, HS, or HB), over a one-hour period, and followed a semi-structured guide. Written notes were taken during the focus groups, these were used to produce a thematic analysis.

### 2.3. Ethics

This study was submitted to the University of Nottingham Faculty of Medicine and Health Sciences Research Ethics Committee. This committee reviewed study details and confirmed that ethical approval was not required (ref: FMHS 510-0322) due to it being preparatory work towards a future research programme. Those taking part in the survey and focus groups were given information about the study, reassured that any data collected would be anonymised, and were free to withdraw at any point. They were also given the opportunity to ask any questions after receiving the initial information before deciding to take part.

## 3. Results

### 3.1. Mapping Exercise

There were a total of 76 responses to the survey from nature-based activity providers. When the survey closed, the link to the online survey link had been viewed 209 times, representing an estimated participation rate of 36%. However, it is unknown how many of these were people who then went on to click through again later and complete.

Of the 76 respondents, 8 did not offer nature-based activities and therefore were excluded from the rest of the survey, leaving 68 full responses. There were no missing data as responses were mandatory for all required questions before submission was possible. For free text questions, responses varied in length, but all respondents offered the necessary information. The survey was mostly completed by the project leads/senior staff (50%, *n* = 34) or project officers (19%, *n* = 13).

The vast majority of the nature-based activities were designed to promote mental health and wellbeing in those attending (94%, *n* = 64). Responses were from across the East Midlands regions of the UK but concentrated within the Derbyshire and Nottinghamshire counties. This was an expected finding as both Derbyshire and Nottinghamshire received government funding in 2021 to develop green prescribing in these areas. Therefore, these areas had established networks of nature-based providers that could be utilised for distributing the survey.

Nature-based activities were well established in the region, with half of activities/organisations running for over 5 years (Table 1). Most organisations did not charge any fees to attendees (54%, *n* = 37), and many others subsidised the nature-based activity to make it accessible to those who could not pay (Table 1). For those who did charge fees, these varied depending on the activity from <£1 to £100 per session (e.g., part funded forest schools to more costly equine experiences).

#### 3.1.1. Types of Activity

**Figure 1:**
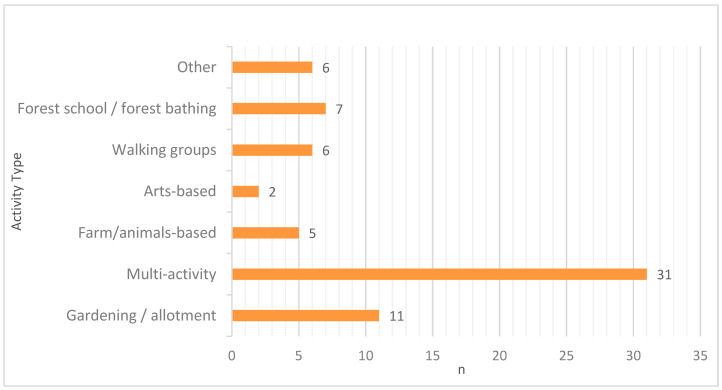
Types of nature-based activities.

Many of the organisations offered more than one type of activity within their natural settings (46%, *n* = 31), such as gardening, outdoor cooking, arts and crafts, and mindfulness/yoga (Figure 1). Other activities offered included forest schools or forest bathing, walking groups, arts-based activities, farm/animal activities, and gardening/allotments (Figure 1). Most of the nature-based activities were based within the organisation’s own natural setting (e.g., gardens and woodland), However, some (especially walking groups) made use of publicly available areas to conduct activities. Some of the activities were aimed at specific groups, such as those with mental health issues or in certain locations. However, many did not restrict access and were open to all those who felt they could benefit. An illustrative example of a walking group aimed to reduce social isolation and improve mental health through organised entry level group walks, including buggy walks for parents. To illustrate an organisation offering multiple activities, one community allotment and forest offered children’s activities (and forest school), a lunch club, grow-your-own projects, yoga, and adult learning courses.

#### 3.1.2. Governance, Evaluation, and Funding

Most of the organisations stated that they evaluated their nature-based activities in some way (79%, *n* = 54). This was either completed internally or with external partners such as universities. For some this was quite informal, by gathering testimonials and monitoring numbers attending. Others collected data through established outcome measures (e.g., wellbeing scales) and/or through case studies, and many collected follow-up data with attendees over time.

Most organisations appeared to have definite (41%, *n* = 28) or potential (37%, *n* = 25) plans to scale up their nature-based activity demonstrating an appetite for development and growth in this area. However, difficulties were noted in terms of acquiring longer-term funding, availability and training of staff or volunteers, and levels of interest from the community. Some organisations also saw the increasing interest in green prescribing as an opportunity for expansion, others described it as not being helpful due to the lack of funding and referrals.

**Figure 2:**
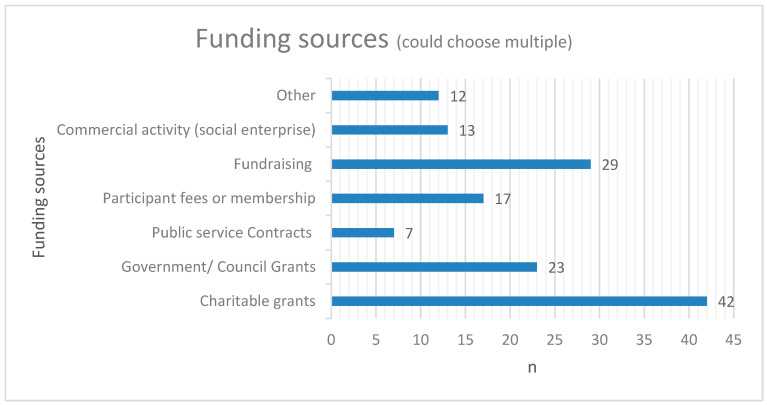
Funding sources of projects/organisations.

Almost half of the organisations stated that their nature-based activities were at risk of closing (49%, *n* = 33). The main reasons offered for this was due to lack of, short-term, or uncertain funding. Other reasons included small numbers of attendees, disruption caused by the coronavirus (COVID-19) pandemic, inappropriate or limited referrals from social prescribers, and a lack of volunteers to help run the activities. Organisations had a mix of funding models (Figure 2), with some including income from social enterprise/commercial activities or fees from attendees. However, many organisations were reliant on charitable grants, fundraising, and funds from government bodies to run their activities.

#### 3.1.3. Access and Attendance

Attendees could gain access to the nature activities through a range of different routes, with some organisations happy for people to arrive on the day without prior booking (Table 2). Other routes of access included via social or green prescribing link workers, other professional referrals, or self-referral (Table 2). There was also variety in the amount of people that accessed projects each year. Some were quite small organisations or nature-based activities with participation numbers of 0–49 per year (22%, *n* = 15), but many catered for larger numbers with 34% (*n* = 23) of projects having more than 200 people per year accessing activities (Table 2). The vast majority of organisations did not set any limits on how many times people could attend activities, with many stating ‘as long as they want’. A few, however, were dependent on levels of funding, and some mentioned that activities were seasonal and closed over winter.

#### 3.1.4. Pregnancy and Future Research

Just over half of the organisations stated that their nature-based activities were already available to young pregnant women or those with new babies (53%, *n* = 36). Only four organisations commented that their activities were not suitable for this group (6%), with reasons such as the physical nature of the work, or organisations feeling that there was a risk other clientele (e.g., prison leavers, those suffering with addiction, high mental health needs) may not be compatible with pregnant women. Many other organisations welcomed the idea of adapting the activities to suit young pregnant women before and after birth. Considerations included making sure there were appropriate facilities, accessing referrals for this group, funding, risk assessments, and the physical nature of the activities. Most organisations also answered ‘yes’ (71%, *n* = 48), or ‘maybe’ (25%, *n* = 17) when asked if they were happy to be contacted about a future research project to evaluate nature-based interventions with young pregnant women. Only 4% (*n* = 3) of organisations stated that they would not like to be contacted, demonstrating that most organisations had an interest in adding to the evidence base around nature activities and their impact on health and wellbeing.

### 3.2. Women’s Focus Groups

Four focus groups were completed with young women (*n* = 11). Sharing demographics was optional, five young women opted to share details and described their ethnicity as White British (*n* = 3), Black/Black British Caribbean (*n* = 1), and Black/Black British African (*n* = 1). Education attainment ranged from A levels or post-secondary vocational qualifications to university (first degree). Women were aged 22–25 years and had been pregnant within the last 2 years, there was a mix of first-time mothers and those who had more than one child.

#### 3.2.1. Experiences and Perceptions of Nature-Activities

The women were asked about their experiences and views of engaging in nature activities during pregnancy. There was a mix of women who had engaged in nature informally or through groups, and those that had not spent much time in nature during pregnancy. The women were positive about the activities and felt strongly that being outdoors and in nature was beneficial to both mental and physical health during and after pregnancy. Another benefit identified of being outdoors, e.g., going for walk, was that there were more opportunities for social interactions which were helpful during what was described as a very lonely time. This included chance encounters with people they already knew and brief interactions with others. These encounters also avoided the pressure of socialising if individuals were shy or socially anxious. Having opportunities to socialise and a reason to ‘get out of the house’ during and after pregnancy was an important aspect to the young women. Some women described losing most of their friends during pregnancy as they were no longer able to do the activities that were part of their lives prior to pregnancy (e.g., social activities that included alcohol or late nights out). Group-based nature activities were viewed as a very positive opportunity to make new friends with peers and expand support networks. Some women also mentioned that they would welcome meeting people of different ages as they lacked parental figures in their lives. Others suggested that same age might be better due to the stigma of young pregnancy.

#### 3.2.2. Types of Activities

The women suggested a variety of activities that would be appealing to them, including walking, yoga, gardening, meditation, outdoor cooking, music, arts and crafts. Some women expressed that being with animals would be helpful but acknowledged that it could present a health risk to pregnant women. The young women felt that engaging in an activity might help remove some of the social pressure when meeting new people. The women agreed that mid-pregnancy may be the best time to start a group activity and that it would be most beneficial to continue throughout pregnancy and beyond into the first few months with a new baby. Activities around once a week or fortnight appeared to be most appealing in terms of frequency for nature groups, with a duration of 1–2 h.

#### 3.2.3. Perceived Challenges and Solutions

The women raised key challenges to consider when providing nature activities, these centred on transport, facilities, and anxieties about attending. It was important for some that activities were accessible by public transport. Others were anxious about public transport and preferred something walkable or accessible by car. A need was highlighted for appropriate facilities to be available, such as toilets and baby changing areas. It was felt that some women may be anxious about attending a new group. Suggestions to combat this included being able to bring someone to accompany them; having detailed information available about the activities so that they know exactly what to expect; introduction or endorsement by a health professional (e.g., midwife or health visitor); and connecting women by social media or messaging prior to sessions starting. The women articulated that communication of the potential mental health benefits of being in nature would encourage people to attend. The groups felt that the best ways to recruit women for future research and interventions would be through midwives, health visitors, family nurses, and social media (particularly Facebook).

### 3.3. Provider Focus Groups

Two focus groups were held with nature activity providers to explore their experiences of operating nature-based group activities. Four main themes were identified, these are detailed below, and Table 3 provides illustrative notes from the focus group supporting these.

#### 3.3.1. Impact of Nature-Based Activities

The providers described their perceived benefits of nature-based activities, such as learning skills, increasing self-esteem, and participants being able to lose themselves in activities rather than focusing on worries or anxieties. There was also a focus on the positive impact of being in a natural environment and connecting with nature. Providers agreed that long-term interventions were most likely to have the highest impact on health and wellbeing, but most felt that there may be measurable benefits after 6–12 weekly sessions.

#### 3.3.2. Barriers and Facilitators to Activity Provision

They described challenges with providing their nature-based activities such as the availability and funding of (e.g., public) transportation, particularly for rural sites. They stated that attendance could be an issue, particularly due to the anxiety of some clients of attending new group. Suggestions to improve attendance included high quality communications (pamphlets, text messaging, and in-person) to reduce participant anxiety, and over recruitment to allow for attrition. Providers also recommended that expectations are managed with regard to the types of facilities available, suitable clothing and footwear, and the weather.

#### 3.3.3. Experiences of Green Prescribing Systems

Providers were asked about their experiences of green prescribing systems. For most providers, their experiences of this had been mainly negative, particularly in terms of lack of funding for their services and few or inappropriate referrals. Some providers felt they were able to create more positive experiences in the green prescribing system through workarounds such as by-passing social prescribers, or being very selective about which referrals they could take.

#### 3.3.4. Nature-Based Activities and Pregnancy

When asked about taking part in nature activities during pregnancy, providers felt there would be minimal changes to current services and risk assessments. However, it was noted that some isolated sites may have challenges related to toilet and baby change facilities which were currently very basic. Providers expressed that involvement in nature-based activities during pregnancy could also have positive impacts on women’s parenting styles and their children’s relationship with nature.

## 4. Discussion

This research shows that there are a wide variety of nature-based activities available for green prescribing programmes in the East Midlands region of the UK. The vast majority of these activities were perceived by the women and nature-activity providers as being suitable for women during pregnancy, and there was strong interest in the young women for participating in these.

This study found that obtaining adequate funding was one of the biggest issues for nature-activity providers. Both in the mapping survey and focus groups, providers expressed their disappointment that no funding was provided through green and social prescribing to those that deliver the services. This is in line with previous work detailing funding models in the UK, where although funding is being provided by NHS England for social prescribing programmes, this is not directed to the community organisations providing the services [38]. Instead, this funding is for one ‘link worker’ per primary care network—a group of general practitioner (GP) practices for populations of around 30–50,000 people [38]. The link worker role is to connect people referred by GPs to local support (e.g., nature-based activities, befriending services, etc.) and has been described as key to the social prescribing process [39]. However, our findings were that this left many community organisations and nature-activity providers having to find funding elsewhere, mainly through fundraising and applying for charitable grants. Many of the providers we spoke to were dissatisfied with this funding model and also with other green prescribing processes such as obtaining adequate and appropriate referrals from link workers. These types of functionality and sustainability issues have been referred to as a key knowledge gap in green prescribing which requires further empirical investigation [40]. These findings are also useful for the delivery of social prescribing programmes in general, particularly for developing policy and practice in these systems.

The vast majority of nature-based activities found in our mapping survey were designed to promote mental wellbeing. Providers further described the mechanisms of this in the focus groups, particularly in terms of increasing self-esteem and the mindfulness of tasks taking focus away from worries and anxieties. This reflects existing evidence from other studies of social prescribing where it is described as providing a more holistic approach to health and support, by re-building service users’ self-confidence, skills and self-reliance [41,42]. A recent systematic review of nature prescriptions (green prescribing) has demonstrated that nature-based interventions have a moderate to large effect on improving depression and anxiety scores [43]. This review also supports our finding that nature-activities take many different forms and involve a wide range of activities, calling for more research on how different modes of delivery may affect outcomes [43]. The providers in the focus groups believed that any time doing nature-based activities could have a positive effect on mental health and wellbeing but agreed that longer-term activities of at least 6–12 weeks should show a measurable effect. This is in line with previous research, which found that the optimum duration of nature sessions was 8–12 weeks [1]. The women contributing to focus groups also commented on the dose and duration of sessions, agreeing with providers that long-term interventions were best but felt benefits immediately as well. The women suggested that a duration of 1–2 h per session would be most appropriate to offer benefits to women whilst still allowing for other commitments. This concurs with a previous systematic review of nature-based interventions suggesting an optimum dose of 20–90 min [1]. These findings have implications for theories of change in the broader field of mental health promotion, whether delivered as part of green prescribing services or not. The findings also add evidence about the importance of long-term access to green space, which can inform placemaking policies as to the role nature can play in supporting wellbeing.

Both women and providers stressed the importance of social aspects of nature-based interventions. For young women, pregnancy could be a very lonely time due to no longer being able to join in with former peer activities. A current systematic review suggests that accessing green space may have a positive effect on loneliness, but this is based on low-quality evidence with a need for more high-quality experimental designs [44]. Although there are very few randomised trials in this area, one reported a significant decrease in stress and loneliness among low-income parents following park prescriptions but did not find any additional benefits for parents in groups compared to individuals, however, this was a small study with only three group outings [45]. Qualitative synthesis of social prescribing has described reduced loneliness and feeling a sense of belonging in a community [42]. However, some drawbacks to social prescribing were also found where people did not feel like they fit in with the group or did not like the other participants or facilitator [42]. More research on the connection between green prescribing and social isolation or loneliness is required, particularly focusing on women and pregnancy.

Practical aspects of nature-based interventions were raised by women and providers as being important to facilitate participation and adherence to an intervention. This echoes the findings of a realist review of social prescribing programmes, where accessibility of the activities in terms of cost, travel, or equipment needed, affected how likely participants were to attend sessions [39]. This review also agrees with our findings that participants require reassurance to attend a first session through processes such as detailed information, regular contact, and being accompanied by someone else [39]. Husk et al., further suggest that the skill of the facilitators is important to maintain attendance [39].

The main strength of this study is the mixed methods approach which gives an overview of the types of nature-based activities available in a region, the views of providers from running these activities, and the acceptability of these activities to young pregnant women. This work is limited by small samples in the young women’s focus groups, due to some women not attending as planned. This may have influenced the discussion and increases the likelihood of some seldom heard voices not being included. However, the women who participated were from a range of backgrounds and holding both in-person and online focus groups increased the reach of the recruitment. It is possible that not all community organisations will have been reached in the mapping survey. However, the snowballing aspect of sampling increases the likelihood of reaching the most active organisations in local communities. This research was also limited by being based in one geographic region of the UK (East Midlands); however, this area is broadly representative of the UK, including both urban and rural areas, with a mix of different socio-economic groups.

## 5. Conclusions

Poor mental health and wellbeing during pregnancy can have long lasting effects on women, children, and families. Green and social prescribing is an approach that is spreading rapidly across the UK and internationally, However, there is limited high-quality experimental evidence of its effectiveness in specific groups, such as young pregnant women. This study has taken a mixed methods approach to determine what type of nature-based activities are available for green prescribing programmes, and adds insight into organisational aspects such as funding, longevity of services, and routes of accessing services. This novel work also adds a new perspective—the provider’s experiences of running nature activities and their interactions with green prescribing systems, which is not available in the wider literature. Focus groups with young women have provided further insights to inform future research into nature-based interventions with young pregnant women, particularly in terms of acceptability.

Future research should include robustly designed randomised controlled trials to increase the evidence base for green prescribing programmes. There is also a need for testing the feasibility of implementing nature-based activity programmes during pregnancy, and particularly for young pregnant women. This study offers detailed information about the priorities and needs of both young women and the third sector providers to support intervention development for a feasibility trial. A feasibility trial is required to test methods for recruitment, randomisation, and rates of attrition to support a future fully powered randomised controlled trial testing nature-based interventions for promoting the mental wellbeing of young pregnant women. The findings from this study can also guide green prescribing policy development due to the unique focus on the experiences and needs of third sector providers of nature-based activities.

## Figures and Tables

**Table 1:** Nature-based activity (organisation) details.

Length of Time Project Established	*n* (%)	Fee Charging to Attendees	*n* (%)
<1 year	9 (13%)	Yes	13 (19%)
1–2 years	15 (22%)	No	37 (54%)
2–5 years	10 (15%)	Sometimes	18 (27%)
5–10 years	11 (16%)		
>10 years	23 (34%)		

**Table 2:** Accessing nature-based activities.

Number of People Accessing Nature Activity per Year	*n* (%)	Activity Access Route	*n* *
0–49	15 (22%)	Via Social/Green Prescribing Link Workers	38
50–99	15 (22%)	Via other professional referral (e.g., GP, mental health services)	32
100–149	10 (15%)	Self-referral	45
150–199	5 (7%)	Informal—just turn up	40
200+	23 (34%)	Other	18

* Percentages not presented as multiple routes could be chosen.

**Table 3:** Provider focus groups—themes and illustrative notes.

Theme	Illustrative Notes
Impact of nature-based activities	▪ Makes significant difference—environment/green space lifts spirits. ▪ Can support skills development and build resilience. ▪ Work with hands tunes in to body—“losing self” in craft. ▪ Use of veg from garden- helps understanding of how fresh food is good for body. ▪ Long-term benefits: that there is a community; there is a group that can help; equipping people with coping mechanisms. ▪ Relaxation and mindfulness- encourages “stepping back”; useful for pregnant women and new mothers due to significant life change. ▪ Sessions run in blocks of 6—by week 3 you can notice a difference. ▪ Longer-term intervention if possible. However, 6 weeks would suffice to change outcomes/get results. More likely to get buy-in if 6 weeks than 12. ▪ Having another focus beyond the specific reasons for attendance (pregnancy, anxiety, etc.). ▪ Can help to reduce health inequalities.
Barriers and facilitators to activity provision	▪ Location- not accessible by public transport. ▪ Achieving attendance; sign ups not following through. ▪ Financial support for transport. ▪ Small groups work better to help people be comfortable and engaged and can be creative—activity can take the pressure off needing to talk so good to have a balance. ▪ Important to send out lists of suggested clothing for activities—some people may not have waterproof clothing or the resources to buy them. ▪ Funding to provide basic food and drink is important.
Experiences of green prescribing systems	▪ Had some good and some inappropriate referrals. ▪ Organisations may work with a specific group and green prescribing may refer clients with different needs causing problems in considering needs of whole group. ▪ Being very specific on publicity materials about client group and inviting social prescribers to taster days can help with referral quality. ▪ No finance attached to green prescribing; third sector does not receive anything to support infrastructure/running costs. ▪ Layers of management, and no further contact with them or support. ▪ Feels like a tick box exercise rather than something that has been carefully considered. ▪ Not enough information is provided by the link worker about the individual’s issues (from the GP). ▪ Not over reliant on (social prescribing) link workers as they don’t usually have much information. Some are helpful, some are not. Using self-referrals to become independent from them.
Nature-based activities and pregnancy	▪ Educating young women but also looking at impacts on their children—do they transfer to their parenting and use green space for their children? ▪ Being able to focus on the mother is important—bringing other children would take focus away. Offers an oasis, an escape. ▪ Bad weather could be an issue—keeping warm. ▪ Brief medical form or disclaimer may be required. ▪ Terrain-pushchairs possibly challenging if poor weather. ▪ Toilet facilities are basic—no hot water.

## Data Availability

Data is not available. However, a list of nature-based activities located in the mapping work is available at https://www.nottsmaternity.ac.uk/documents/nature-in-pregnancy/list-of-nature-based-providers-in-em.pdf (accessed on 1 October 2023).

## Supplementary Materials

The following supporting information can be downloaded at: https://www.mdpi.com/article/10.3390/ijerph20206921/s1, Supplementary File S1: Survey instrument.

## References

[1] Coventry P.A., Brown J.V.E., Pervin J., Brabyn S., Pateman R., Breedvelt J., Gilbody S., Stancliffe R., McEachan R., White P.C.L. (2021). Nature-based outdoor activities for mental and physical health: Systematic review and meta-analysis. SSM-Popul. Health.

[2] Callaghan A., McCombe G., Harrold A., McMeel C., Mills G., Moore-Cherry N., Cullen W. (2021). The impact of green spaces on mental health in urban settings: A scoping review. J. Ment. Health.

[3] White M.P., Pahl S., Wheeler B.W., Depledge M.H., Fleming L.E. (2017). Natural environments and subjective wellbeing: Different types of exposure are associated with different aspects of wellbeing. Health Place.

[4] Thomas F. (2015). The role of natural environments within women’s everyday health and wellbeing in Copenhagen, Denmark. Health Place.

[5] Pritchard A., Richardson M., Sheffield D., McEwan K. (2020). The Relationship Between Nature Connectedness and Eudaimonic Well-Being: A Meta-analysis. J. Happiness Stud..

[6] Mitchell R., Popham F. (2008). Effect of exposure to natural environment on health inequalities: An observational population study. Lancet.

[7] Müller-Riemenschneider F., Petrunoff N., Yao J., Ng A., Sia A., Ramiah A., Wong M., Han J., Tai B.C., Uijtdewilligen L. (2020). Effectiveness of prescribing physical activity in parks to improve health and wellbeing—The park prescription randomized controlled trial. Int. J. Behav. Nutr. Phys. Act..

[8] Foster A., Thompson J., Holding E., Ariss S., Mukuria C., Jacques R., Akparido R., Haywood A. (2021). Impact of social prescribing to address loneliness: A mixed methods evaluation of a national social prescribing programme. Health Soc. Care Community.

[9] Kotera Y., Richardson M., Sheffield D. (2022). Effects of Shinrin-Yoku (Forest Bathing) and Nature Therapy on Mental Health: A Systematic Review and Meta-analysis. Int. J. Ment. Health Addict..

[10] British Medical Association (BMA) (2018). Addressing Unmet Needs in Women’s Mental Health. https://www.bma.org.uk/media/2115/bma-womens-mental-health-report-aug-2018.pdf.

[11] NHS Digital (2016). Adult Psychiatric Morbidity Survey: Survey of Mental Health and Wellbeing, England, 2014. https://digital.nhs.uk/catalogue/PUB21748.

[12] Howard L.M., Khalifeh H. (2020). Perinatal mental health: A review of progress and challenges. World Psychiatry.

[13] Lee D.T.S., Chung T.K.H. (2007). Postnatal depression: An update. Best. Pract. Res. Clin. Obstet. Gynaecol..

[14] Ghimire U., Papabathini S.S., Kawuki J., Obore N., Musa T.A. (2021). Depression during pregnancy and the risk of low birth weight, preterm birth and intrauterine growth restriction—An updated meta-analysis. Early Hum. Human. Dev..

[15] Bauer A., Knapp M., Parsonage M. (2016). Lifetime costs of perinatal anxiety and depression. J. Affect. Disord..

[16] Netsi E., Pearson R.M., Murray L., Cooper P., Craske M.G., Stein A. (2018). Association of Persistent and Severe Postnatal Depression With Child Outcomes. JAMA Psychiatry.

[17] Biaggi A., Conroy S., Pawlby S., Pariante C.M. (2016). Identifying the women at risk of antenatal anxiety and depression: A systematic review. J. Affect. Disord..

[18] Dennis C.L., Falah-Hassani K., Shiri R. (2017). Prevalence of antenatal and postnatal anxiety: Systematic review and meta-analysis. Br. J. Psychiatry.

[19] Fawcett E.J., Fairbrother N., Cox M.L., White I.R., Fawcett J.M. (2019). The Prevalence of Anxiety Disorders During Pregnancy and the Postpartum Period: A Multivariate Bayesian Meta-Analysis. J. Clin. Psychiatry.

[20] Shorey S., Chee C.Y., Ng E.D., Chan Y.H., San Tam W.W., Chong Y.S. (2018). Prevalence and incidence of postpartum depression among healthy mothers: A systematic review and meta-analysis. J. Psychiatr. Res..

[21] Siegel R.S., Brandon A.R. (2014). Adolescents, Pregnancy, and Mental Health. J. Pediatr. Adolesc. Gynecol..

[22] Wong S.P.W., Twynstra J., Gilliland J.A., Cook J.L., Seabrook J.A. (2020). Risk Factors and Birth Outcomes Associated with Teenage Pregnancy: A Canadian Sample. J. Pediatr. Adolesc. Gynecol..

[23] Liu H., Ren H., Remme R.P., Nong H., Sui C. (2021). The effect of urban nature exposure on mental health—A case study of Guangzhou. J. Clean. Prod..

[24] McEachan R.R.C., Prady S.L., Smith G., Fairley L., Cabieses B., Gidlow C., Wright J., Dadvand P., van Gent D., Nieuwenhuijsen M.J. (2016). The association between green space and depressive symptoms in pregnant women: Moderating roles of socioeconomic status and physical activity. J. Epidemiol. Community Health.

[25] Islam M.Z., Johnston J., Sly P.D. (2020). Green space and early childhood development: A systematic review. Rev. Environ. Health.

[26] Lumber R., Richardson M., Sheffield D. (2017). Beyond knowing nature: Contact, emotion, compassion, meaning, and beauty are pathways to nature connection. PLoS ONE.

[27] McEwan K., Richardson M., Sheffield D., Ferguson F.J., Brindley P. (2019). A smartphone app for improving mental health through connecting with urban nature. Int. J. Environ. Res. Public Health.

[28] Greenblatt M., Becerra R.M., Serafetinides E.A. (1982). Social networks and mental health: An overview. Am. J. Psychiatry.

[29] Anderson K., Laxhman N., Priebe S. (2015). Can mental health interventions change social networks? A systematic review. BMC Psychiatry.

[30] Elsenbruch S., Benson S., Rücke M., Rose M., Dudenhausen J., Pincus-Knackstedt M.K., Klapp B.F., Arck P.C. (2007). Social support during pregnancy: Effects on maternal depressive symptoms, smoking and pregnancy outcome. Hum. Human. Reprod..

[31] Morikawa M., Okada T., Ando M., Aleksic B., Kunimoto S., Nakamura Y., Kubota C., Uno Y., Tamaji A., Hayakawa N. (2015). Relationship between social support during pregnancy and postpartum depressive state: A prospective cohort study. Sci. Rep..

[32] Racine N., Madigan S., Plamondon A., Hetherington E., McDonald S., Tough S. (2018). Maternal adverse childhood experiences and antepartum risks: The moderating role of social support. Arch. Womens Ment. Health.

[33] Oakley A., Hickey D., Rajan L., Rigby A.S. (1996). Social support in pregnancy: Does it have long-term effects?. J. Reprod. Infant. Psychol..

[34] Grady M.A., Bloom K.C. (2004). Pregnancy outcomes of adolescents enrolled in a Centering Pregnancy program. J. Midwifery Womens Health.

[35] Megnin-Viggars O., Symington I., Howard L.M., Pilling S. (2015). Experience of care for mental health problems in the antenatal or postnatal period for women in the UK: A systematic review and meta-synthesis of qualitative research. Arch. Womens Ment. Health.

[36] Price A., Janssens A., Dunn-Morua S., Eke H., Asherson P., Lloyd T., Ford T. (2019). Seven steps to mapping health service provision: Lessons learned from mapping services for adults with Attention-Deficit/Hyperactivity Disorder (ADHD) in the UK. BMC Health Serv. Res..

[37] Eysenbach G. (2004). Improving the quality of Web surveys: The Checklist for Reporting Results of Internet E-Surveys (CHERRIES). J. Med. Internet Res..

[38] Sandhu S., Alderwick H., Gottlieb L. (2022). Financing Approaches to Social Prescribing Programs in England and the United States. Milbank Q..

[39] Husk K., Blockley K., Lovell R., Bethel A., Lang I., Byng R., Garside R. (2020). What approaches to social prescribing work, for whom, and in what circumstances? A realist review. Health Soc. Care Community.

[40] Robinson J.M., Breed M.F. (2019). Green Prescriptions and Their Co-Benefits: Integrative Strategies for Public and Environmental Health. Challenges.

[41] Cooper M., Flynn D., Avery L., Ashley K., Jordan C., Errington L., Scott J. (2023). Service user perspectives on social prescribing services for mental health in the UK: A systematic review. Perspect. Public. Health.

[42] Liebmann M., Pitman A., Hsueh Y.C., Bertotti M., Pearce E. (2022). Do people perceive benefits in the use of social prescribing to address loneliness and/or social isolation? A qualitative meta-synthesis of the literature. BMC Health Serv. Res..

[43] Nguyen P., Astell-Burt T., Rahimi-Ardabili H., Feng X. (2023). Effect of nature prescriptions on cardiometabolic and mental health, and physical activity: A systematic review. Lancet Planet. Health.

[44] Astell-Burt T., Hartig T., Gusti Ngurah Edi Putra I., Walsan R., Dendup T., Feng X. (2022). Green space and loneliness: A systematic review with theoretical and methodological guidance for future research. Sci. Total Environ..

[45] Razani N., Morshed S., Kohn M.A., Wells N.M., Thompson D., Alqassari M., Agodi A., Rutherford G.W. (2018). Effect of park prescriptions with and without group visits to parks on stress reduction in low-income parents: SHINE randomized trial. PLoS ONE.

