# Supplementary material for: Nature-Based Interventions in the UK: A Mixed Methods Study Exploring Green Prescribing for Promoting the Mental Wellbeing of Young Pregnant Women
Source: Int J Environ Res Public Health. 2023 Oct 13;20(20):6921. doi: 10.3390/ijerph20206921 (PMC10606054; doi:10.3390/ijerph20206921)
Supplement: Supplementary file 1 [file ijerph-20-06921-s001.zip › ijerph-2599611-supplementary.pdf]

Supplementary File S1 - Survey instrument

1. Do you offer nature-based activities in the East Midlands for adults and/or young people? *

- ◯ Yes
- ◯ No

2. Is the nature activity designed to promote mental health and wellbeing? *

- ◯ Yes
- ◯ No

3. Where in the East Midlands is your project based? *

You can select more than one area if applicable

- ☐ Derbyshire
- ☐ Leicestershire
- ☐ Lincolnshire
- ☐ Northamptonshire
- ☐ Nottinghamshire
- ☐ Rutland

4. What is your role in this initiative/project? *

- ◯ Project lead / Senior staff
- ◯ Project Officer / Project Management role
- ◯ Administrator
- ◯ Volunteer
- ◯ Other

5. What is the name of your organisation and nature-based initiative/project? *

6. If you have a website or facebook page, please add it here.

7. Can you please describe the nature-based initiative/project in as much detail as possible? *

If your organisation runs several distinct projects (e.g. a community allotment and guided nature walks), please fill out the survey again for each project if possible.

8. How long has this initiative/project been running? *

- Less than a year
- 1-2 years
- 2-5 years
- 5-10 years
- 10 years +

9. Do you charge a fee to participants? *

- Yes
- No
- Sometimes

10. If so, please briefly explain what fees are charged

11. How is this initiative/project funded? *

- ☐ Charitable grants
- ☐ Local authority / NHS / Government Grants
- ☐ Local authority / NHS / Government Contracts (contracted to provide a service)
- ☐ Participant fees or membership
- ☐ Fundraising
- ☐ Commercial activity (e.g. in social enterprises)
- ☐ Other

12. Do you evaluate the impact of this initiative/project or measure particular outcomes? *

- ○ Yes
- ○ No

13. If so, please briefly describe how you evaluate your service and/or how outcomes are measured

e.g. do you measure things like: number of people attending, what people do or don't like about the initiative, how people feel after participating, any long-term effects to wellbeing.

14. Are there any plans to scale up this initiative/project? *

◯ Yes

◯ No

◯ Maybe

15. Please briefly describe your plans to scale up

16. Is this initiative/project at risk of closing due to lack of funding (or other reasons)? *

◯ Yes

◯ No

17. If so, please briefly describe how the project is at risk

18. How many people access this project per year? *

This question refers to this specific nature activity, not how many people your organisation/service reaches as a whole.

◯ 0-49

◯ 50-99

◯ 100-149

◯ 150-199

◯ 200+

19. Is this initiative/project restricted to certain groups of people? *

e.g. by postcode, health diagnosis, gender or age?

20. Can people keep attending the project for as long as they want, or is there a set number of visits per person? *

Also please mention if the project is seasonal and therefore has a natural cut off point.

21. How do people access this initiative/project? *

- [ ] Via Social/Green Prescribing Link Workers
- [ ] Via other professional referral (e.g. GP, mental health services)
- [ ] Self-referral
- [ ] Informal - just turn up on the day
- [ ] Other

22. Is this a project that is already available to young pregnant women and/or those with new babies? *

- ( ) Yes
- ( ) No

23. If no, please describe whether you feel this project could be adapted to include young pregnant (and/or postnatal) women in the future.

24. Do you know of any other nature-based projects or networks to promote mental health and wellbeing in the East-Midlands?

If so, please can you provide publicly available contact information (e.g. website or social media) so we can find out more.

25. Are you happy for the project name, organisation name, and website/facebook page to be made publicly available in our list of nature-based activities in the East Midlands? *

All other details and answers you have provided will be kept confidential.

- Yes
- No

26. Would you be happy to be contacted about a future research project to evaluate nature-based social activities for promoting wellbeing in young pregnant women? *

If your initiative/project could be made available this group

- Yes
- No
- Maybe

27. Please provide a contact name and email address to discuss further without commitment

This content is neither created nor endorsed by Microsoft. The data you submit will be sent to the form owner.
